# Quality of life in enuretic children

**DOI:** 10.1590/S1677-5538.IBJU.2020.0308

**Published:** 2020-12-20

**Authors:** Raquel A. Rangel, Carolina Ribeiro Seabra, Carlos Eduardo P. F. Ferrarez, Josana L. Soares, Mauro Choi, Robert Gomes Cotta, Andre Avarese de Figueiredo, José de Bessa, Jose Murillo B.

**Affiliations:** 1 Universidade Federal de Juiz de Fora Seção de Urologia Departamento de Cirurgia Juiz de ForaMG Brasil Departamento de Cirurgia, Seção de Urologia - Universidade Federal de Juiz de Fora - UFJF, Juiz de Fora, MG, Brasil

**Keywords:** Chil, Nocturnal Enuresis, Quality of Life

## Abstract

**Introduction:**

Nocturnal enuresis is a highly incident chronic disorder that generates countless problems to the child and their parents. Bed-wetting has significant negative impacts on self-esteem and the performance of children. The aim of the current study is to assess the quality of life of enuretic children, as well as its association to sex and age.

**Patients and Methods:**

Thirty-nine enuretic children (23 boys) and 49 healthy children (27 boys) without any history of previous treatment for enuresis or voiding dysfunction were included. Age ranged between 6 and 11 years old. The “AUQEI” questionnaire was applied in a private environment to all children by the same researcher (psychologist) to evaluate quality of life.

**Results:**

Enuretic children displayed loss in quality of life when compared to non-enuretic (35.9% of enuretic x 16.3% of non-enuretic, p=0.035). They were mostly affected in their daily activities (p=0.02). No significant differences were found in the association of sex and gender with quality of life. These results suggest that, children with nocturnal enuresis have 2.87 times more chances of having loss in quality of life compared to non-enuretic.

**Conclusions:**

Enuresis has a great impact in quality of life of children. This impact is not related to the age or sex of the child.

## INTRODUCTION

Enuresis, according to the International Children's Continence Society, is defined as the micturition that occurs inappropriately during sleep by a child older than 5 years old whose age should have already allowed such vesical control to have been reached (1).

Enuresis, popularly known as “bed-wetting”, is highly frequent, being diagnosed with a higher prevalence among boys than girls aged 6-13 years (2, 3). It is more common among children in families with history of enuresis and low education level, decreasing the prevalence with age (3). It is a problem that afflicts thousands of children throughout the World affecting the development, self-esteem and performance of the individual, as well as the relationship within and outside of the family (4). Being a chronic disorder, it generates problems to the children and their families (5). Its continuance may cause a high level of stress, negatively affecting self-esteem (6), which may affect their QoL (QoL). Many enuretic children will present behavioral and emotional problems and, when relevant, psychological evaluation and treatment are recommended (7).

Definitions of QoL are as numerous as the methods of evaluating them and there is no consensus about its meaning. In the scientific literature, there are numerous theoretical and methodological controversies, mainly due to the multidimensional and subjective concept, but also by the numerous factors that influence in its construction as the object of evaluation or research (8),^9^).

Studies on QoL in adults have progressed substantially, whereas there are few studies focusing on QoL in children. The interest in studying QoL in children has only increased in the past decade (10). Few studies have addressed QoL in children presenting enuresis. “Given that a major motivation in contemporary society is for individuals to experience a life of quality, QoL should be adopted as the universal outcome towards which all our efforts regarding children ultimately should be directed” (10).

We hypothesized that children presenting with enuresis are more likely to have a decrease in their quality of life. Therefore, the current study aims to contribute to fill this gap and evaluate the QoL in those children.

## MATERIAL AND METHODS

This case-control study included 88 children that were divided into 2 groups, Study Group (SG) including thirty-nine children suffering from primary monosymptomatic enuresis seen at our Enuresis Clinics, and Control Group (CG) including 49 healthy children with the same background and controlled by age and gender, interviewed at public schools in our area. The classification of primary monosymptomatic enuresis includes those children that have never being dry during the night for a period of six months or more and have no other symptoms rather than enuresis.

To exclude those with daytime voiding dysfunction (non-monosymptomatic enuresis) and those with secondary enuresis in the study group, a thorough medical history was taken. In non-enuretic children (CG), the past medical history was also gathered to exclude previous treatment for enuresis or voiding dysfunction. It was also excluded those, either enuretic and non-enuretic, with other chronic diseases, and history of previous treatment or undergoing treatment for enuresis.

The study was approved by the university research ethics committee (Protocol #: 1283.329.2007) and all parents or legal guardians of the participating children signed an informed consent.

To assess QoL the AUQEI (Autoquestionnaire Qualité De Vie Enfant Imagé) questionnaire (11), validated in Brazil in the year 2000 by Assumpção (12), was applied to all children. Its current version consists of 26 questions or areas that explore family relationships, social activities, health, bodily functions and separation, 18 of them contained in 4 dimensions or factors: 1) Daily Activites: issues relating to activities at school, meals, bedtime, and going to a medical appointment (questions 1, 2, 4, 5, 8); 2) Family: issues related to their opinion about their parents, their familie's opinions about them, and their opinions about themselves (questions 3, 10, 13, 16, 18); 3) Recreation/Leisure: issues related to vacation, birthday and relationship with grandparents (questions 11, 21, 25); 4) Autonomy: issues related to independence, relationships with friends and school evaluation (questions 15, 17, 19, 23, 24). The questionnaire is answered by the child, and the answers to the questions are either “very unhappy”, “unhappy”, “happy” and “very happy”, having the values of 0, 1, 2 and 3, respectively. The sum of all the questions generates a score with a maximum of 78. Scores below 48 are considered as loss in QoL (11, 12). AUQUEI has an internal reliability estimate by alpha Cronbach coefficient of 0.87 for global evaluation and also for each domain. In addition, each item is more correlated to its sub-domain than to the others.

During the first appointment at the Enuresis Clinic, a multidisciplinary team (pediatric urologist, nurse, and psychologist) evaluated the child. During the psychological interview, the AUQEI questionnaire was applied individually and the child answered it with the help of the researcher. After the initial evaluation, all participating children were followed in the clinic by all member of the team.

For the control group, the researcher went to public schools and after authorization of the school director and from the parents, the AUQEI questionnaire was applied to children with matched age and sex to the study group, chosen at random. The answering of the questionnaire was carried out with each child individually and in a reserved room.

The primary endpoint of the study was to evaluate if children with enuresis present impaired QoL and as secondary endpoints we evaluated if gender or age influence in QoL. For a significance level (alpha) of 0.05 (two-tailed) and a 80% power to detect a change of 25% the sample size calculated was 40 patients with enuresis with a proportion of 1:1.25 for the control group (50 patients). Association analyses between study and control groups were done through a contingency Table (2×2). For the association with sex, QoL, enuresis, the Chi-Square test was used. To assess the association between age and QoL it was used the Student's t-test. Values of p <0.05 were considered significant.

## RESULTS

A total of 88 children were included in the study, being 39 in SG, presenting monosymptomatic primary enuresis (23 boys - 59%), and 49 in CG - non-enuretic children (27 boys - 55.1%).

Of the 50 boys analyzed, 23 (46%) were enuretic, while of the 38 girls, 16 (42.1%) were enuretic (p=0.716) (Table-1). The average age in the non-enuretic group was 8.41±1.41 years of age (6 to 11) and in the enuretic group 8.26±1.57 years of age (6 to 11) (p=0.635) (Table-1).

**Table 1:** Distribution of the children included in the study according to gender and age

	Gender - n (%)		Total	p-value
	Boys	Girls		
CG	27 (55.1%)	22 (44.9)	49 (100%)	
SG	23 (59%)	16 (41%)	39 (100%)	
Total	50 (56.8%)	38 (43.2%)	88 (100%)	0.716
	**Age years**			**p-value**
CG	8.56±1.45	8.23±1.38		
SG	8,52±1.56	7.73±1.49		
**Total**	**8.41±1.41**	**8.26±1.57**		**0.635**

**Figure 1:**
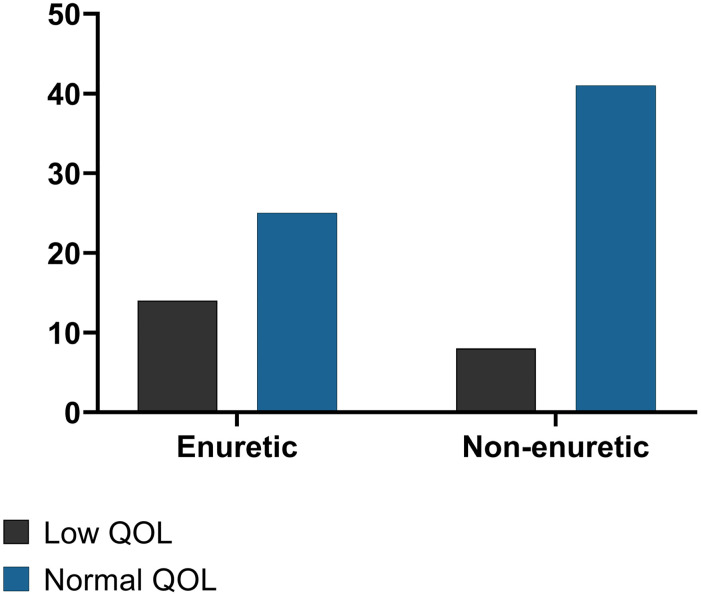
Quality of Life in enuretic children compared to non-enuretic ones (p=0.035).

The analysis of the AUQEI questionnaires of enuretic children showed that 14 of them (35.9%) were faced with loss in QoL (score bellow 48), while in the non-enuretic group, only 8 displayed such loss (16.3%) (p=0.035) (Figure-1). The median score in SG was 49 (41)-68) while in CG it was 51 (43)-65) (p=0.035) (Table-2). Children with enuresis presented 2.87 more chances of having loss in QoL compared to non-enuretic (OR: 2.87 [1.061-8.069] 95% CI).

**Table 2:** Evaluation of QOL according to each domain of AUQUEI.

Domain	Group	Median	25% Percentile	75% Percentile	p-value
Quality of Life (Total)	SG	49.0	46.0	52.0	0.035
	CG	51.0	52.0	56.5	
Autonomy	SG	6.0	5.0	7.0	0.34
	CG	6.0	5.0	7.0	
Daily Activities	SG	9.0	7.0	10.0	0.02
	CG	10.0	9.0	11.0	
Recreation/Leisure	SG	8.0	7.0	9.0	0.30
	CG	8.0	6.0	9.0	
Family	SC	11.0	9.0	13.0	0.64
	CG	11.0	10.0	12.0	

Only daily activities (function) presented difference between the groups (0.02) when all domains of AUQUEI were evaluated separately (Table-2).

No difference was found when comparing gender and QoL, being 50 [48-56] for boys and 50 [47-54] for girls (p=0.742). The same was true for age and QoL, where no correlation was found (r=0.148; p=0.167).

The present study demonstrates that children with enuresis have lower QoL when compared to normal ones, with 2.87 times more chances of loss in their QoL than those non-enuretic when evaluated with the use of the AUQEI questionnaire.

The AUQEI questionnaire was validated for assessing QoL in children (11),^12^) younger than 12 years of age and was chosen for this study because it is a generic instrument that makes the comparison possible between QoL of children afflicted by a disease and those who are healthy.

Butler et al. (13) states that aside from psychosocial problem, disruptive experiences in the childhood apparently enhance the vulnerability to enuresis. Moreover, the same author affirms that enuretic children go through perplexity, humiliation, social isolation, fear of being discovered and having the feeling of immaturity. The parents of enuretic children also suffer the impact of the problem (14),^15^), usually relating the cause of enuresis to deep sleep (16). Furthermore, studies suggest that enuretic children have poor quality of sleep, which could indicate that sleep disorders can impact even more their QoL (17).

A study by Moffat et al., demonstrated that children that underwent enuresis treatment improved the Piers-Harris Self-Concept Scale (a scale to monitor change in self-concept over time and has six sub-scores: behavioral adjustment, intellectual and school status, physical appearance and attributes, freedom from anxiety, popularity, and happiness and satisfaction), especially those who had larger decrease in wet nights, when compared with those not treated (18). Monosymptomatic enuresis may also have a negative impact in the child's perception of the quality of attachment although caregivers reported no significant dissociative behavior (19). In addition, Mota et al. showed an important association between enuresis and psychiatric disorders in children at 6 and 11 years age. Enuretic children showed higher prevalence of any psychiatrist, hyperactivity and oppositional disorders compared with those without enuresis (20).

The analysis of our data showed significant loss in QoL of enuretic children when compared to those non-enuretic (p=0.035). It can also be observed from the results that over one third (35.9%) of the enuretic children displayed a reduction in QoL, meaning 2.87 times more chances of having loss in their QoL compared to non-enuretic. Evaluating each domain of the AUQUEI we observed that the most affected domain was that of daily activities (function), which means that it impacts their day by day life, and not impairing their relationship with their families, leisure and autonomy.

On the other hand, in the non-enuretic group a high percentage of loss in QoL was also found. A 16% prevalence of loss in QoL, in the control group, is possibly related to low socio-economic level of the children. These findings are in agreement of those of Schast et al., that, although not only for monosymptomatic enuretic children, found that those suffering from lower urinary symptoms have an impairment in their QoL, especially those with day and night time symptoms (21). In counterpart, Natale et al., found that, in children with daytime urinary incontinence, health related QoL was significantly reduced in their parent's rating but not in child's rating (22). In agreement with these results, Nelli et al., 2019, have demonstrated that enuresis had a significant impact in QoL of children presenting sickle cell disease (23).

Our group have demonstrated that children suffering from enuresis may be subject of punishment in up to 100%, including all kinds of punishment, from verbal to physical aggression, especially in those families with history of enuresis and lower educational level (24). This data may be associated as one of the cause effects in the loss of QoL of these children. Therefore, this suggests that it would be adequate to provide counseling to these children and families, as it is suggested by Houts (25), who says that, the bigger the psychological contact, the bigger will the benefits be. In our Enuresis Clinic, we observe that children helped by the psychological team tended to have better and faster treatment results. Adding the evaluation of QoL when evaluating children with enuresis may help screening those children that are suffering a greater impact from their enuresis and to propose different approach with a closer psychological follow-up.

The likelihood of having enuresis among boys and girls varies among different studies (26),^27^). A study in our country has showed no statistically significant difference between boys and girls (28). In agreement with the similar incidence of enuresis in both genders, the evaluation of the impact of enuresis in QoL showed an absence of association with gender. One also would expect that older children would suffer more from having enuresis since they have a more active social life and are more worried about aspects related to their bodies and their behavior. It has been shown that as age increases a worsening in self-esteem occurs (16). In the opposite of this thought, this study found no difference between those children older and younger than 9 years of age. The age of 9 years old was chosen because is in the midline between 6 and 11 years of age in the children included in this study.

This present study has some limitations. The number of patients is small, but even though was sufficient to demonstrate the differences in QoL between those with and without enuresis. Although AUQEI covers a lot of aspects it is somewhat subjective, which could impair the results. Other factors that could affect QoL were not studied and further studies with uni- and multivariate analysis need to be done to better investigate this matter in conjunction with enuresis.

Studying QoL related to a specific disease may be difficult due to other social, cultural, economic and familiar aspects that can interfere in this subject. The data presented herein using a matched population strongly suggests that those children with enuresis face a worsening in their QoL. These findings are important for families and physicians dealing with enuretic children because their disease is frequently seen as a minor problem, therefore been neglected and left aside, and its consequences may affect the present and in many cases the future of these children.

## CONCLUSIONS

In conclusion, this study suggests that enuretic children have low QoL when compared to non-enuretic ones. This finding reinforces the need of a reliable therapeutic approach early in life, with a multidisciplinary group to prevent them from this loss. Gender and age did not influence in QoL in this population.
